# IDEIS: a tool to identify PTPRC/CD45 isoforms from single-cell transcriptomic data

**DOI:** 10.3389/fimmu.2024.1446931

**Published:** 2024-10-09

**Authors:** Juraj Michalik, Veronika Niederlova, Ondrej Stepanek

**Affiliations:** Laboratory of Adaptive Immunity, Institute of Molecular Genetics of the Czech Academy of Sciences, Prague, Czechia

**Keywords:** PTPRC, CD45, isoform, alternative splicing, immunology, T cell, single-cell RNA sequencing, gene expression

## Abstract

Single-cell RNA sequencing (scRNA-seq) methods are widely used in life sciences, including immunology. Typical scRNA-seq analysis pipelines quantify the abundance of particular transcripts without accounting for alternative splicing. However, a well-established pan-leukocyte surface marker, CD45, encoded by the *PTPRC* gene, presents alternatively spliced variants that define different immune cell subsets. Information about some of the splicing patterns in particular cells in the scRNA-seq data can be obtained using isotype-specific DNA oligo-tagged anti-CD45 antibodies. However, this requires generation of an additional sequencing DNA library. Here, we present IDEIS, an easy-to-use software for CD45 isoform quantification that uses single-cell transcriptomic data as the input. We showed that IDEIS accurately identifies canonical human CD45 isoforms in datasets generated by 10× Genomics 5’ sequencing assays. Moreover, we used IDEIS to determine the specificity of the *Ptprc* splicing pattern in mouse leukocyte subsets.

## 1 Introduction

Recently, robust single-cell RNA sequencing (scRNA-seq) methods have emerged to study gene expression at the single-cell level, and have become a widely used tool in life sciences (1). In immunology, scRNA-seq is widely used for the quantification and analysis of known subsets and states of immune cells, as well as for the identification of new ones (2, 3). The typical scRNA-seq analysis pipeline quantifies the abundance of transcripts of individual genes but does not extract information concerning their alternative splicing, which could be present in the data as well.

CD45 (encoded by the *PTPRC* gene) is a receptor tyrosine phosphatase that is a well-established pan-leukocyte surface marker. Moreover, individual lymphocyte subsets have different patterns of CD45 splicing (4, 5), which is commonly addressed by flow cytometry phenotyping panels using splicing-sensitive antibodies (6). *PTPRC* is located on chromosome 1 in both human and murine genomes. *PTPRC* contains over 30 exons, out of which exons 4, 5, and 6 (encoding parts of the protein called A, B, and C, respectively) are alternatively spliced (7). This leads to various protein isoforms named according to the exons included, such as CD45RABC (all three alternative exons included) or CD45RO (all three alternative exons excluded).

Human naïve T cells express CD45RA (CD45 isoform expressing exon A), which is replaced by CD45RO upon activation and differentiation into memory/effector T cells (8, 9). Terminally differentiated T cells re-express CD45RA and are called effector-memory CD45RA^+^ T cells (T_EMRA_) (10). Murine naïve, but not activated, T cells express CD45RB (11). A variant of CD45, called CD45R, or B220, is used as a marker of murine B cells (12, 13).

The expression of CD45 isoforms can be identified in scRNA-seq experiments using a method called CITE-seq. It is based on labeling cell surface proteins with specific antibodies conjugated with DNA oligonucleotides, which can be subsequently sequenced and matched with the corresponding cell transcriptomes (14). However, this approach requires the generation and sequencing of an additional DNA library, which is associated with a prolonged experimental time and additional costs.

Here, we describe and validate a software for the efficient detection of CD45 isoforms directly from transcriptomic scRNA-seq data, called identification of isoforms (IDEIS). It is tailored for the (re)analysis of scRNA-seq data generated using 10× Chromium Single Cell 5’ kits without CITE-seq labeling of CD45 isoforms.

## 2 Methods

### 2.1 Technical aspects of IDEIS software

The IDEIS software uses a 10× Cell Ranger (15) BAM file as an input. The conversion to FASTQ was performed using a 10× Genomics bamtofastq 1.4.1. The option ‘*--locus*’ of this software is used to extract only reads that overlap the *PTPRC* locus, with exact limits of locus depending on the organism and the reference. The software samtools 1.14 was used during the BAM pre-processing step to identify the GTF file formatting (16, 17). The mapping and quantification steps are performed using Salmon Alevin 1.9.0 (18). The conversion of salmon alevin output to RDS and 10X-compatible count matrix was performed using R script (19).

The IDEIS software was tested with 10× Cell Ranger versions 3.0.2 and 5.0.1.

### 2.2 Data availability and pre-processing

All the data used in this project were downloaded from publicly accessible databases. In total, four different single-cell datasets were analyzed (**Supplementary Table 1**). The first three datasets, from papers of the Collora et al., Yu et al., and Mogilenko et al. were obtained from Gene Expression Omnibus of National Center for Biotechnology Information (GEO NCBI) (20) under respective accession numbers GSE187515 (21), GSE212998 (22), and GSE145562 (23). The last dataset, used in the paper of Lawlor et al., was obtained from the European Nucleotide Archive (ENA) and is available under the accession number PRJEB40376 (24). The Mogilenko et al. dataset contained murine data, whereas the rest of the datasets included human samples.

For each dataset, we used only the relevant samples as specified in **Supplementary Table 2**. For the Mogilenko et al. dataset, the BAM files and count matrices directly provided by the authors were downloaded from GEO NCBI and used for IDEIS and downstream analyses. For other datasets, we downloaded the FASTQ files for gene expression and CITE-seq and mapped them using 10× Cell Ranger software (15) to generate BAM files and count matrices for each sample separately. For the Collora et al. dataset, we used Cell Ranger 3.0.2 for mapping to human reference provided by 10× (version 3.0.2), while for the Yu et al. and Lawlor et al. datasets, we mapped the reads with 10× Cell Ranger 5.0.1 on human reference created from files corresponding to build GRCh38 downloaded from Ensembl version 102 (25). For Collora et al., Yu et al., and Lawlor et al. datasets, the list of mapped antibodies is provided in **Supplementary Table 1**.

The analyses with IDEIS were performed on BAM files either provided directly by the authors (the Mogilenko et al. dataset) or generated by us from their FASTQ files (the Collora et al., Yu et al., and Lawlor et al. datasets). The BAM for the Collora et al. and Yu et al. datasets were also pre-filtered to reads from cells that passed the initial quality control. Since the Lawlor et al. dataset provided FASTQ files that contain reads R2 of two different lengths, the BAM files generated by Cell Ranger were split by length using samtools and option –e ‘*length(seq)<=100*’ or ‘*length(seq)>100*’ and analyzed by IDEIS for each length separately. Final counts were generated by the addition of counts from both groups.

### 2.3 Comparison of IDEIS and CD45er

We analyzed the BAM files for both the Corolla et al. and Yu et al. datasets using CD45er according to official instructions (https://github.com/getzlab/10x-cd45-isoform-quantification) (26). CD45er outputs probabilities for each read aligning to CD45 to belong to one of the isoforms, RAX, RABX, RBX, RBCX, RABCX, or RX. The total probability of *CD45RA* was calculated as the sum of probabilities of reads belonging to isoforms RAX, RABX, and RABCX. The probability of *CD45RO* corresponds to the value marked in the column RX. We then generated the final counts used in further downstream analysis as an aggregation of these probabilities using cellular barcodes.

### 2.4 Data analysis

All data were analyzed using R 4.2.1 (19) with Seurat 4.1.1 (15). All parameters were chosen to generate the best visualization and clustering, which would allow the definition of cellular subtypes and remove eventual contaminations. Initially, basic analysis was performed for each sample. To retain only cells with good sequencing depth, the minimum number of features was set to 1,000 for the Lawlor et al. dataset and 200 for other datasets. For the Collora et al., Yu et al., and Lawlor et al. datasets, CITE-seq counts were imported as a separate assay and then normalized using the CLR method (27). IDEIS and, if calculated, CD45er results were imported and processed in a similar manner. For each sample, reads mapping to T-cell receptor (TCR)-related genes, as well as ribosomal and mitochondrial genes, were discarded. Each dataset was also subjected to normalization, variable feature detection (1,000 features), scaling, dimensional reduction using PCA (20 principal components were kept), and UMAP and clustering (resolution variable for different datasets; please refer to the code for more details). Clusters of dead cells, contamination of undesired cell types, and any cells with more than 10% reads mapped to mitochondrial genes were removed. Subsequently, samples from the same dataset were integrated using STACAS 2.0.1 (1,000 features for anchors) (28), re-scaled, subjected to dimensional reduction (20 principal components for PCA), and re-clustered (resolution variable for different datasets). In the case of the Mogilenko et al. dataset, the second filtering was performed after integration, followed by another round of post-processing. The datasets were manually annotated using standard markers. The gene expressions visualized on UMAP projections were generated using Seurat-provided functions, with the minimum cut-off for feature expression in all cases being 0. For the Lawlor et al. dataset, the additional maximum cut-off for CITE-seq and IDEIS-computed log_2_-normalized counts of 2 were used.

To compare the performance of CITE-seq or CD45er and IDEIS at single-cell level, we regrouped cells by the number of CD45RA or CD45RO transcripts (numerical interval groups being open on the right) detected by IDEIS and CITE-seq (in which case the counts were scaled to log_10_ + pseudo-count of 1 to avoid non-finite values) or CD45er, calculated the number of cells per group and then plotted as log_10_ + pseudo-count of 1 using a tile plot. The correlation coefficients and p-values were calculated using the original, ungrouped data (log-scaled in the case of CITE-seq).

To compare the results yielded by CITE-seq and IDEIS or CD45er and IDEIS at the level of clusters, we calculated the averages of log_2_-normalized *PTPRC/CD45* isoform counts (IDEIS, CITE-seq) or probabilities (CD45er) per each cluster. We then used these values to compute the Pearson’s correlation coefficient and linear regression between the two approaches.

### 2.5 Subsampling human control CD4^+^ T cell dataset analysis with IDEIS

To simulate the effect of reduced sequencing depth on IDEIS analysis, we employed subsampling of BAM files used as entries for isoform analysis. We subsampled the BAM of each sample from the Collora et al. dataset on percentages ranging from 5 to 100 in increments of 5 with the subsampling option ‘*--subsample*’ of samtools view tool along with ‘*--subsample-seed*’ set to 42. The subset BAM files were then used for analysis by IDEIS, as usual, and the resulting count matrices were imported to Seurat objects as separate assays.

The required sequencing depth was estimated by determining the average length of reads mapping to the genome per cell, which was computed as the number of all reads from experiment mapping to the genome exactly once, divided by the number of cells used in the finalized datasets. Only the reads from these cells were considered. These values were computed separately for each subsampling percentage. We then quantified the amplitude of the effect of subsampling on the quality of IDEIS analysis using two different estimators: i) fraction of cells expressing the given isoform–fraction of cells where the count for the given isoform was above 0; and ii) average log_2_-normalized read count of the entire dataset for each isoform. To determine the maximum theoretical value for both the cases, we fitted the following two-parameter rational function:


$$Y=\;\frac{a\times X}{b+X}$$

where *a* is the maximum value of estimator X, and *b* is the parameter for which Y = 0.5 × a. This function was previously used in a similar case to model the number of somatic single-base mutations as a function of sequencing coverage (29). This model was fitted using the function *nls* from the base R package stats.

### 2.6 Subsampling PBMC T cell dataset analysis with IDEIS

Due to large size of the data, subsampling of the Lawlor et al. dataset was performed only on the Pool 1 sample (sample number SAMEA7463734, see **Supplementary Table 2**). Subsampling was performed separately on the BAM files split by length, as specified previously. The rest of the subsampling process and quantification of its effect were performed as in the case of the Collora et al. dataset.

### 2.7 Benchmarking IDEIS vs CD45er

Benchmarking was performed on the datasets of Collora et al. and Yu et al. Before benchmarking, we subsampled the BAM files by 10× subset-bam to contain only alignments for cells present in whitelists, which were generated from the initial Seurat objects. The execution time was estimated with Unix “time” command and counted as the sum of user and system time (time spent in use mode outside the kernel and in the kernel within the process, respectively). Both software programs were run 40 times, and the results were averaged. Because salmon alevin does not support parallel indexing, indexing was not benchmarked in case of IDEIS. We ran the benchmarking on a cluster with 96 CPUs (Intel(R) Xeon(R) CPU E7-8890 v4, 2.20GHz per CPU) and 1TB RAM.

### 2.8 Code availability

The software is available at https://github.com/Lab-of-Adaptive-Immunity/IDEIS. The code used to process the downloaded datasets, prepare figures as well as running benchmarking is available at: https://github.com/Lab-of-Adaptive-Immunity/IDEIS_data_analysis.

## 3 Results

### 3.1 IDEIS is a software for detection of CD45 isoforms from transcriptomic data

**Figure 1:**
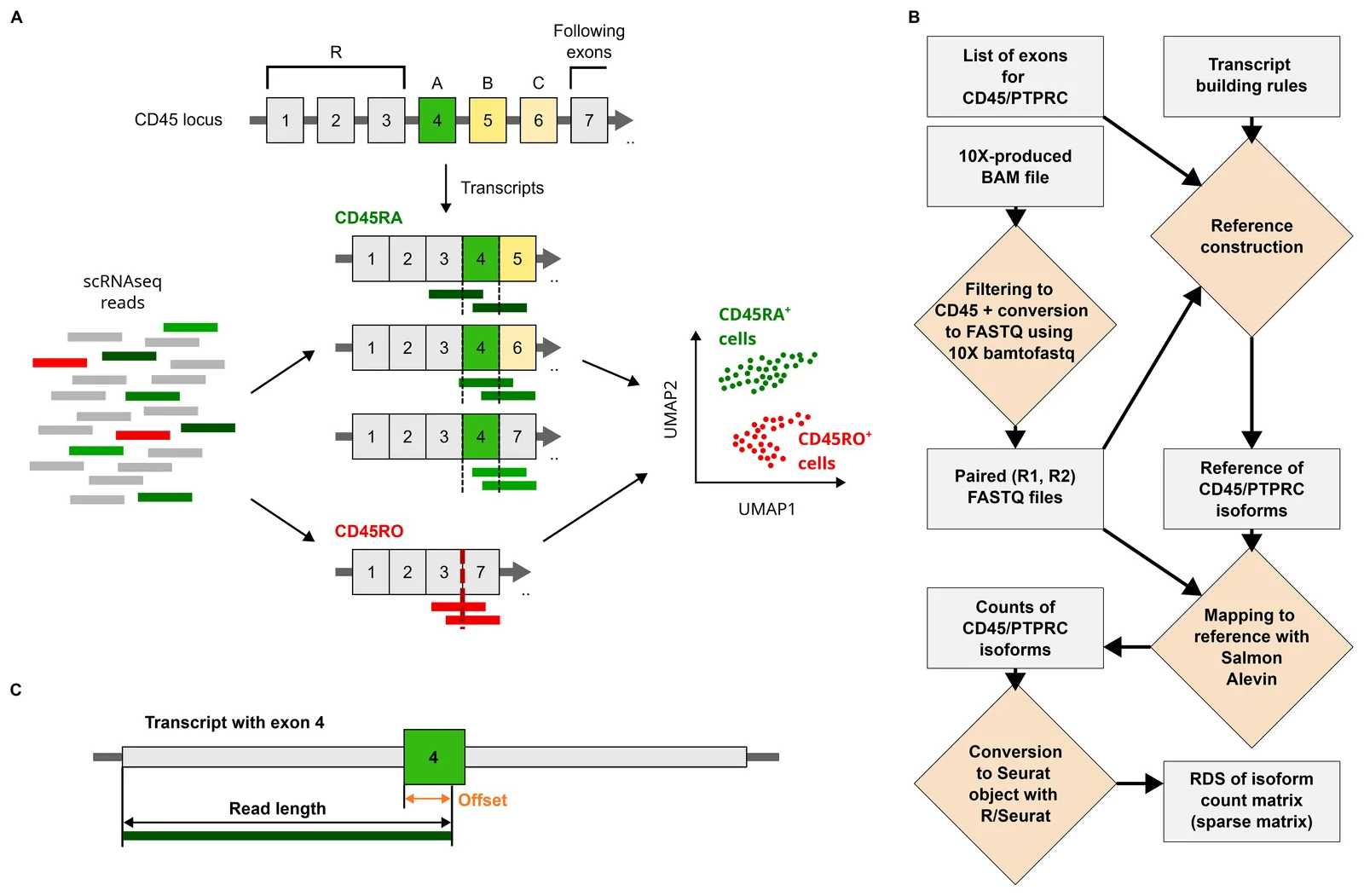
Isoforms of *PTPRC/CD45* and their detection by IDEIS software. **(A)** The principle of identification of *PTPRC/CD45* isoforms using IDEIS software. *PTPRC/CD45* can be transcribed and spliced into a number of variants including *CD45RA* and *CD45RO* isoforms. Some reads generated by sequencing at least partially map to exon A, while others might cover junction between exons 3 and 7 (left and middle). These reads then can be used to identify cells that express *CD45RA* and *CD45RO* isoforms for downstream analysis (right). **(B)** The framework of IDEIS software. Light gray rectangle—object/result, yellow rhombus—process. The first step is finding reads that map to CD45 locus from BAM file produced by 10× Cell Ranger and then dump them into FASTQ files using 10×-bamtofastq software. The length of R2 read is extracted if not provided as parameter, and along with a list of exons and a set of rules providing the information on exons or junctions of interest and the exons around them—transcript building rules—is used to build a reference in such a manner that the reads aligned against it match specific isoforms. The reads from FASTQ files are then mapped against reference using salmon alevin software. The process is finalized by conversion of count matrix generated by salmon alevin into RDS file containing Seurat object. **(C)** The creation of reference used for detection of *PTPRC/CD45* isoforms. The length of reads of R2 is used to generate flanking sequences (orange) to exon of interest, here exon 4 (green) with certain offset. The flanking sequences are built from the sequences of exons following the transcript building rules, which indicate what exons precede or follow exon 4 and in which order/combination. Any read mapping to complete sequence (dark green bar) of transcriptome will also map at least partially to exon 4.

To enhance the annotation of lymphocyte subsets in single-cell data, we created IDEIS, a software program aimed at identifying alternatively spliced variants of the *PTPRC/CD45* gene. The principle of IDEIS software is the creation of a transcriptome reference, to which a read will map only if it contains a specific exon or if it spans a junction of the two neighboring exons. For example, a read mapping to exon 4 identifies *CD45RA* isoform; similarly, a junction between exons 3 and 7 specific to *CD45RO* can be identified by a read that overlaps it (**Figure 1A**).

The reference to which reads are mapped is constructed from adjacent exons or exon–exon junctions; for example, exon 4 can be followed by exon 5, but also by exon 6 or 7 (**Figure 1B**; see also Technical Note in the **Supplementary Material**). All possible links and ordering between exons of interest and possible flanks are exhaustively enumerated by transcript building rules contained within a file with special formatting, which are then used to construct all transcripts that contain an exon of interest or junction with all possible combinations of flanking sequences. The length of these flanks was determined from the length of the reads that were used in the experiment, which was analyzed by IDEIS (**Figure 1C**). The complete reference is therefore made from sequences, each of which contains an exon or a junction of interest, here exons 4, 5, and 6, and the junction between exons 3 and 7, surrounded by a specific set of flanking sequences, which are constructed using information provided by transcript building rules. Flanks shorter than reads ensure that each read that completely maps to a specific reference sequence least partially covers the exon or junction of interest (**Figure 1C**). However, the exhaustiveness of this list ensures that each possible combination of exons is considered.

The software uses 10× Cell Ranger (15) BAM file as input, from which the reads mapping to *PTPRC* locus are extracted and transformed to FASTQ files using 10× bamtofastq script with *‘--locus’* option. These reads were then mapped to the reference, and the desired *PTPRC/CD45* isoforms were quantified using salmon alevin software (18). Finally, the results are transformed into a format that can be easily imported to a Seurat object containing the analyzed dataset as a separate assay.

### 3.2 The performance of IDEIS for human CD4^+^ T cells

**Figure 2:**
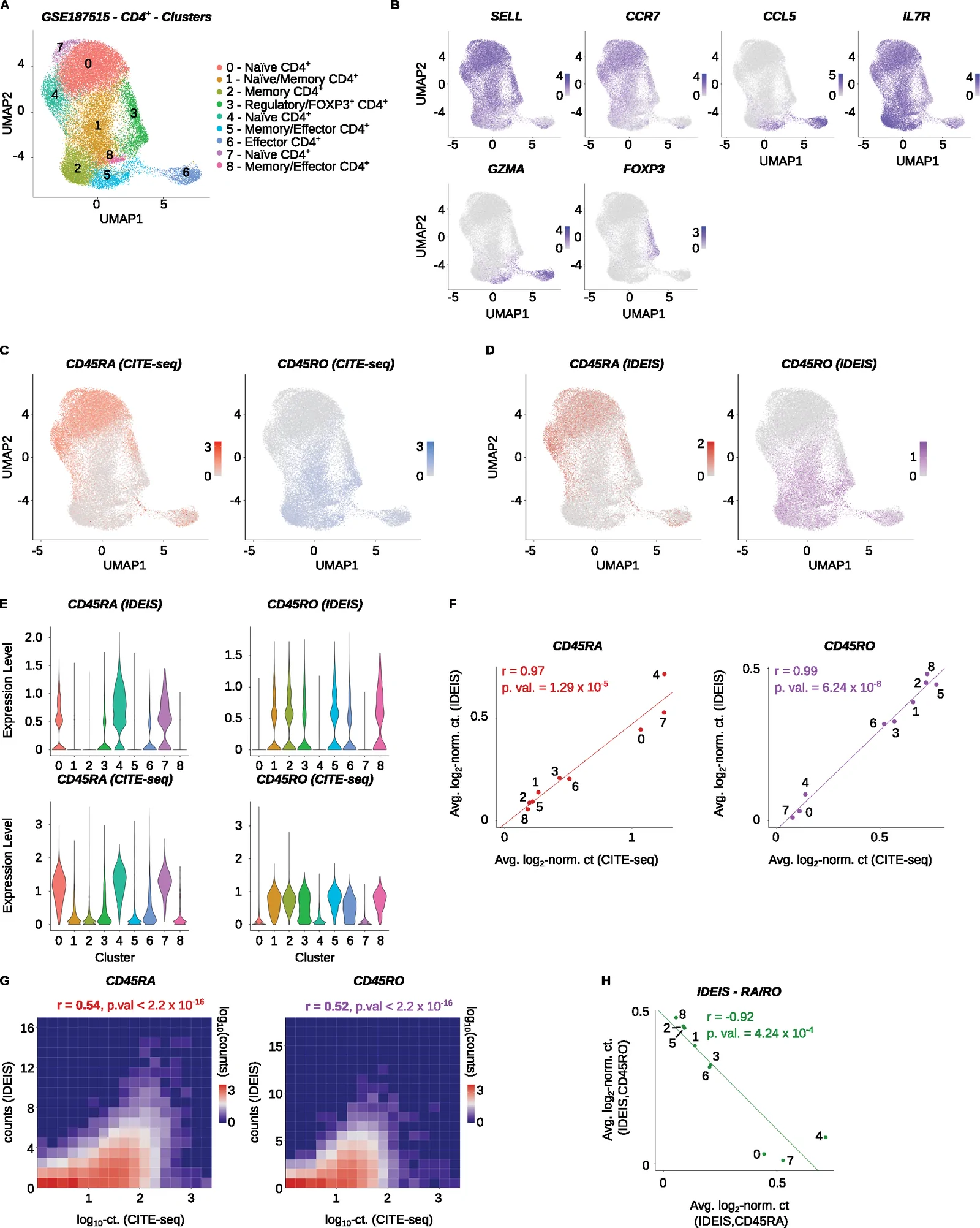
Analysis of *PTPRC/CD45* isoforms of CD4^+^ T cells. Healthy human control samples from the Collora et al. dataset (GSE187515) were analyzed. **(A)** UMAP dimensional reduction and clustering of the finalized dataset, with expert annotations based on the gene expression. The x and y axes show the first and second dimensions of UMAP projection, respectively. **(B)** Visualization of log_2_-normalized expression of selected genes on UMAP projection of the dataset. **(C)** Log_2_-normalized counts obtained by CITE-seq for *CD45RA* (red) or *CD45RO* (blue) isoform on UMAP projection of the dataset. **(D)** Log_2_-normalized counts detected by IDEIS software for *CD45RA* (dark red) or *CD45RO* (purple) isoform on UMAP projection of the dataset. **(E)** Violin plots of normalized expression for *CD45RA* (left column) and *CD45RO* (right column) per cluster as determined by IDEIS (top row) and CITE-seq (bottom row). **(F)** Linear regression and correlation of average log_2_-normalized CITE-seq read counts (x axis) or counts of reads detected by IDEIS software (y axis) corresponding to *CD45RA* (dark red) and *CD45RO* (purple) isoforms for each cluster of the dataset. Each dot represents a value for cluster with numbering as shown on **(A)**. The line shows linear regression curve. r, Pearson’s correlation coefficient, p.val, p-value of slope. **(G)** Tile plot of isoform counts detected by CITE-seq (x axis) and IDEIS (y axis, the values shown correspond to log_10_ with pseudo-count of 1) for *CD45RA* (left) and *CD45RO* (right). The counts per tile are shown in log_10_ scale with pseudo-count of 1. r, Pearson’s correlation coefficient, p.val, p-value of correlation test between cell-wise isoform counts. **(H)** Linear regression and correlation of average log_2_-normalized counts of reads detected by IDEIS software corresponding to *CD45RA* (x axis) and *CD45RO* isoform (y axis). Each dot represents a value for cluster of the dataset with numbering as shown on **(A)**. The line shows linear regression curve. r, Pearson’s correlation coefficient, p.val, p-value of slope.

The location of key exons of *PTPRC/CD45* isoforms is near the 5’ end of the gene; therefore, approaches generating libraries enriched for the 5’ end of the transcript are well suited for CD45 isoform detection. First, we tested the performance of IDEIS software on a dataset published in a study by Collora et al. containing data on CD4^+^ T cells from healthy and HIV-infected patients (Accession Number GSE187515) (21). The gene expression library in this experiment was generated using the Chromium Next GEM Single Cell 5’ Library & Gel Bead Kit v1.1. We only analyzed samples from healthy subjects (**Supplementary Table 2**). The expression levels of *SELL*, *CCR7*, *CCL5*, and *IL7R* characterized distinct CD4^+^ T-cell populations ranging from naïve to memory to effector cells (**Figures 2A, B**). However, data on *CD45RA* and *CD45RO* isoforms are required for further annotation of some cellular subtypes, such as T_EMRA_ cells. CITE-seq using anti-CD45RA and anti-CD45RO antibodies can be used as the gold standard in isoform identification in scRNA-seq data. Therefore, we benchmarked the performance of the IDEIS software with CITE-seq. Both methods identified enrichment of specific isoforms in the same clusters (**Figures 2C–F**). At the single-cell level, CITE-seq was slightly more sensitive than IDEIS, with a significant correlation between the two methods (**Figures 2E, G**).

The analysis of the data generated by IDEIS showed that the expression of *CD45RA* and *CD45RO* was inversely correlated (**Figure 2H**). Furthermore, these data allowed for the identification of some cell subtypes. Notably, cluster 6 showed increased levels of *CD45RA* while expressing effector genes, suggesting that this cluster contained T_EMRA_ cells (**Figure 2A**). In general, IDEAS is a viable alternative to CITE-seq for the identification and annotation of CD45RA/CD45RO expressing clusters, although it is less robust at the single-cell level.

### 3.3 The performance of IDEIS for human CD8^+^ T cells

**Figure 3:**
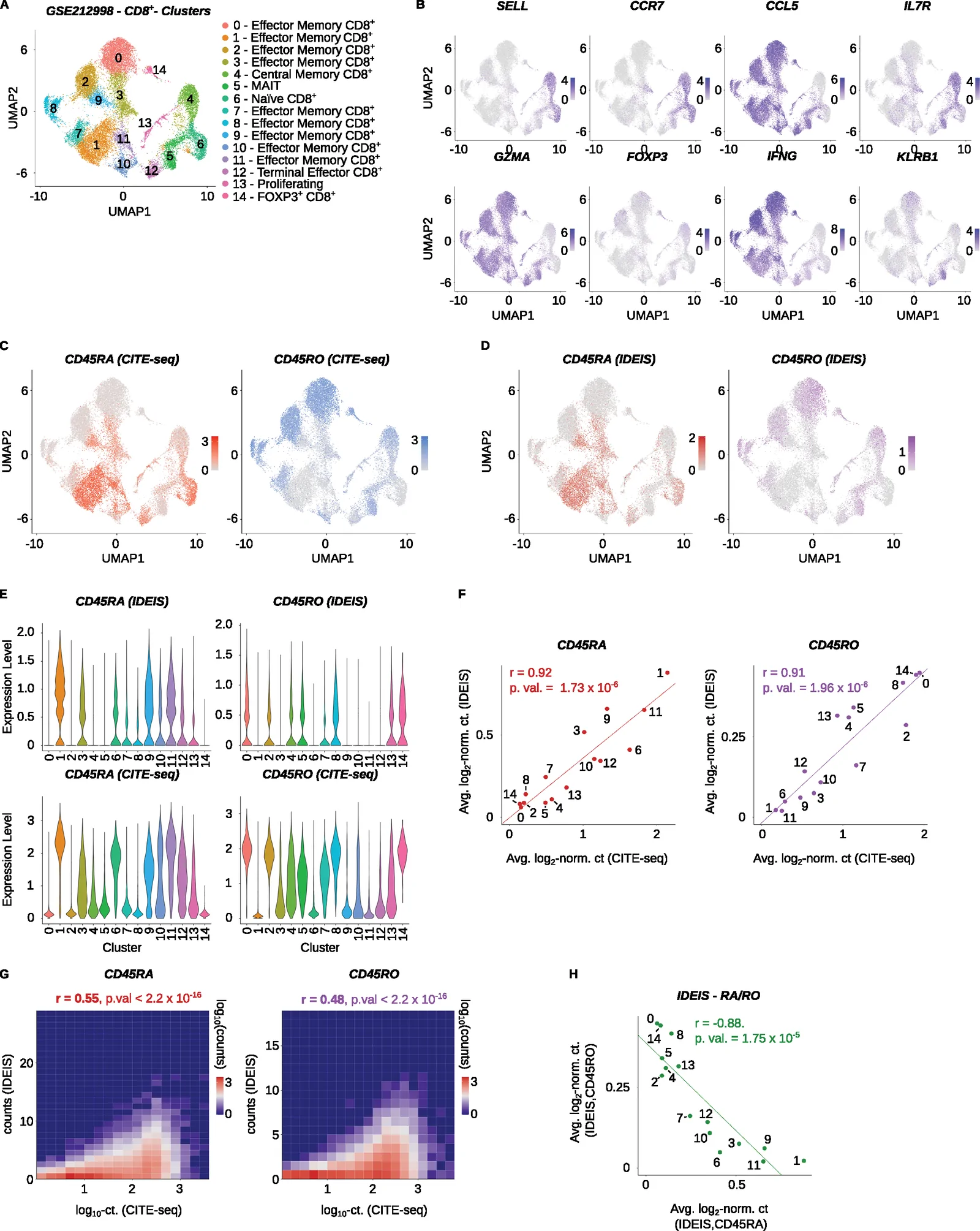
Analysis of *PTPRC/CD45* isoforms of CD8^+^ T cells. CMV-seropositive human samples from the Yu et al. dataset (GSE212998) were analyzed. **(A)** UMAP projection and clustering of the finalized dataset, with expert annotations based on the gene expression. The x and y axes show the first and second dimension of UMAP projection, respectively. **(B)** Visualization of log_2_-normalized expression of selected genes on UMAP projection of the dataset. **(C)** Log_2_-normalized counts obtained by CITE-seq for *CD45RA* (red) or *CD45RO* (blue) isoform on UMAP projection of the dataset. **(D)** Log_2_-normalized counts detected by IDEIS software for *CD45RA* (dark red) or *CD45RO* (purple) isoform on UMAP projection of the dataset. **(E)** Violin plots of normalized expression for *CD45RA* (left column) and *CD45RO* (right column) per cluster as determined by IDEIS (top row) and CITE-seq (bottom row). **(F)** Linear regression and correlation of average log_2_-normalized CITE-seq read counts (x axis) or reads detected by IDEIS software (y axis) corresponding to *CD45RA* (dark red) and *CD45RO* (purple) isoforms for each cluster separately of the dataset. Each dot represents a value for cluster with numbering as shown on **(A)**. The line represents linear regression curve. r, Pearson’s correlation coefficient, p.val, p-value of slope. **(G)** Tile plot of isoform counts detected by CITE-seq (x axis) and IDEIS (y axis, the values shown correspond to log_10_ with pseudo-count of 1) for *CD45RA* (left) and *CD45RO* (right). The counts per tile are shown in log_10_ scale with pseudo-count of 1. r, Pearson’s correlation coefficient, p. val, p-value of correlation test between cell-wise isoform counts. **(H)** Linear regression and correlation of average log_2_-normalized counts of reads detected by IDEIS software corresponding to *CD45RA* (x axis) and *CD45RO* isoform (y axis). Each dot represents a value for cluster of the dataset with numbering as shown on **(A)**. The line shows linear regression curve. r, Pearson’s correlation coefficient, p.val, p-value of slope.

To further validate the performance of IDEIS software at the sell cluster level, we analyzed another dataset (Accession Number GSE212998) (22), which includes CD8^+^ T cells. Gene expression data were generated from CD8^+^ T cells of CMV-seropositive patients profiled using the Chromium Next GEM Single Cell 5’ Kit v2. Distinct clusters of naïve, memory, and effector cells showed expression of their typical markers (**Figures 3A, B**). The *PTPRC/CD45* isoform expression by CITE-seq and IDEIS showed that naïve and some memory/effector T cells expressed *CD45RA*, whereas central memory and some effector memory T cells and MAIT cells expressed *CD45RO* (**Figures 3A, C**). Based on these data, we annotated clusters 1, 3, 9, 11, and 12 (**Figure 3A**) as T_EMRA_ cells.

Both CITE-seq and IDEIS identified *CD45RA* and *CD45RO* isoforms in identical clusters (**Figures 3C–F**). At the single-cell level, the results obtained by CITE-seq showed better performance than by IDEIS, but with a significant correlation between the results (**Figure 3G**). As in the case of CD4^+^ T cells, the expression of *CD45RA* and *CD45RO* in the clusters was strongly anti-correlated (**Figure 3H**). These results demonstrated that IDEIS is suitable for the analysis of human CD8^+^ T cells.

### 3.4 IDEIS does not require a high sequencing depth

**Figure 4:**
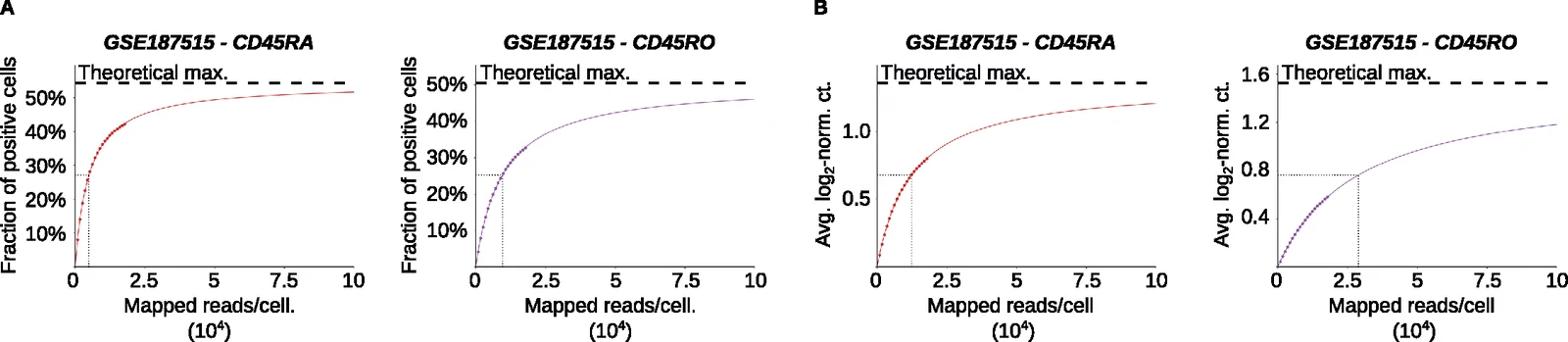
Effect of read subsampling of reads on performance of IDEIS. The Collora et al. dataset (GSE187515) was analyzed. **(A)** The fraction of cells having a positive read count of indicated isoforms identified by IDEIS in function of estimated average number of mapped reads per cell, for *CD45RA* (red) and *CD45RO* (purple) isoforms. The dots show values calculated from subsampled dataset, full colored line shows the fit by two-parameter rational function, and black dashed line shows its asymptote representing maximum theoretically possible value. Black dotted lines show the point where 50% of maximum predicted value has been reached. **(B)** The evolution of average log_2_-normalized read count identified by IDEIS in function of estimated average number of mapped reads per cell, for *CD45RA* (red) and *CD45RO* (purple) isoforms. The meaning of graphical elements is the same as in **(A)**.

Because software performance depends on read mapping to particular parts of the *PTPRC/CD45* transcript, its performance might be strongly influenced by the overall sequencing depth. To address this aspect, we subsampled the BAM files from the Collora et al. dataset to percentages ranging from 5 to 100. To quantify the effects of subsampling on IDEIS performance, we computed the average number of mapped reads per cell as the number of reads mapped at least once divided by the number of cells in the finalized datasets. Furthermore, we computed two different estimators: i) the fraction of cells positive for a given isoform (**Figure 4A**) and ii) the average log_2_-normalized read count for a given isoform (**Figure 4B**). As expected, the number of cells positive for a given isoform, as well as the average log_2_-normalized count of a given isoform, increased with the sequencing depth (**Figure 4A**). We fitted the data with a two-parameter rational function to determine the theoretical maximum and depth with half-maximal performance (**Figures 4A, B**). The half-maximal performance for the determination of the fraction of positive cells achieved at 4,971 reads/cell for *CD45RA* and 9565 reads/cell for *CD45RO* was lower than the actual sequencing depth of the Collora et al. dataset, which was 17,798 reads/cell on average. The half-maximal performance for average log_2_-normalized expression was higher (12,350 for *CD45RA* and 28,940 for *CD45RO*). Overall, IDEIS showed good performance for data with a standard sequencing depth of 15,000-25,000 reads per cell.

### 3.5 IDEIS can be used to identify CD45 in murine T cells

**Figure 5:**
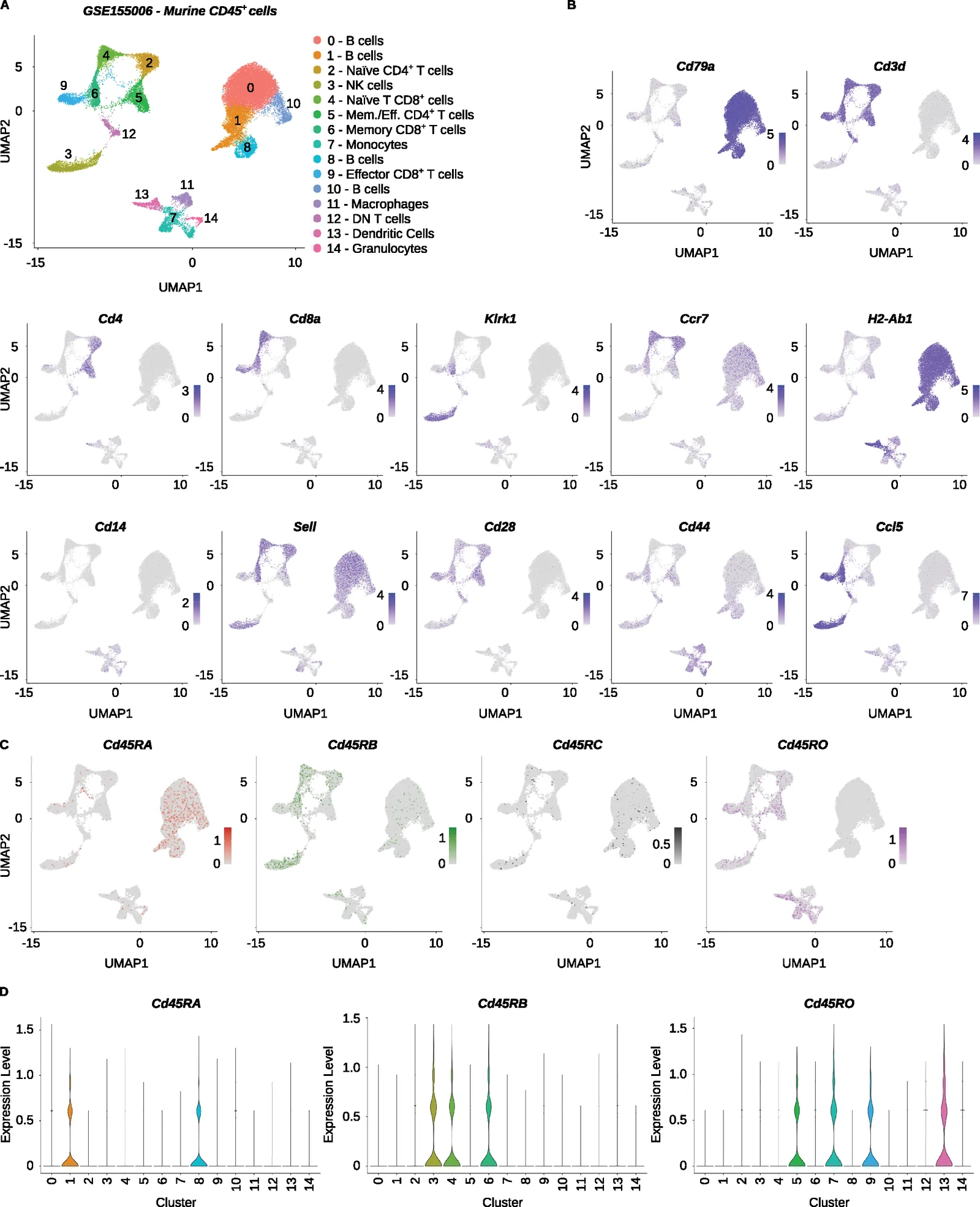
Analysis of CD45 isoforms in murine leukocytes. The Mogilenko et al. dataset (GSE155006) was analyzed. **(A)** UMAP projection and clustering of the finalized dataset, with expert annotations based on the gene expression. The x and y axes show the first and second dimensions of UMAP dimensional reduction, respectively. **(B)** Visualization of log_2_-normalized expression of selected genes on UMAP projection of the dataset. **(C)** Log_2_-normalized counts of reads detected by IDEIS software corresponding to *Cd45RA* (dark red), *Cd45RB* (green), *Cd45RC* (black) and *Cd45RO* (purple) isoforms on UMAP projection of analyzed dataset. **(D)** Violin plots of normalized expression for *Cd45RA* (left), *Cd45RB* (middle) and *Cd45RO* (right) per cluster as determined by IDEIS.

While *PTPRC/CD45* isoforms have been mainly investigated in human cells, the *PTPRC/CD45* splicing pattern differs among T cell subsets in mice as well. Thus, it can be used as an additional parameter for characterizing particular cells in scRNA-seq experiments (9). We analyzed a dataset of murine CD45^+^ cells from multiple organs (Accession Number GSE155006) (23) using IDEIS. The libraries used to obtain these datasets were generated using the Chromium Single Cell 5’ Reagent Kit (the version of the kit not indicated). We clustered and annotated the cells using established markers (**Figures 5A, B**). Since the expression of the *Cd45RB* isoform in murine T cells roughly corresponds to *CD45RA* in humans (11), we included *Cd45RB* and *Cd45RC* isoforms in the analysis (**Figure 5C**). The overall expression of the isoforms identified by IDEIS in mice was lower than that in human samples (**Figure 5D**). *Cd45RA* is mostly expressed in B cells. *Cd45RB* is expressed by naïve and central memory T cells and NK cells. *Cd45RC* showed very low expression in all subsets. The *Cd45RO* isoform is expressed mainly in effector and memory/effector T cells, dendritic cells, and monocytes. Interestingly, naïve CD4^+^ T cells, but not naïve CD8^+^ T cells, express intermediate levels of *Cd45RB* and *Cd45RO*. These results demonstrated for the first time that *PTPRC/CD45* isoforms can be useful for the identification of leukocyte subsets in murine scRNA-seq data. This is not limited to T cell subsets as other immune cell subsets, such as NK cells or monocytes, and also shows characteristic *PTPRC/CD45* splicing patterns.

### 3.6 IDEIS is fast and efficient

**Figure 6:**
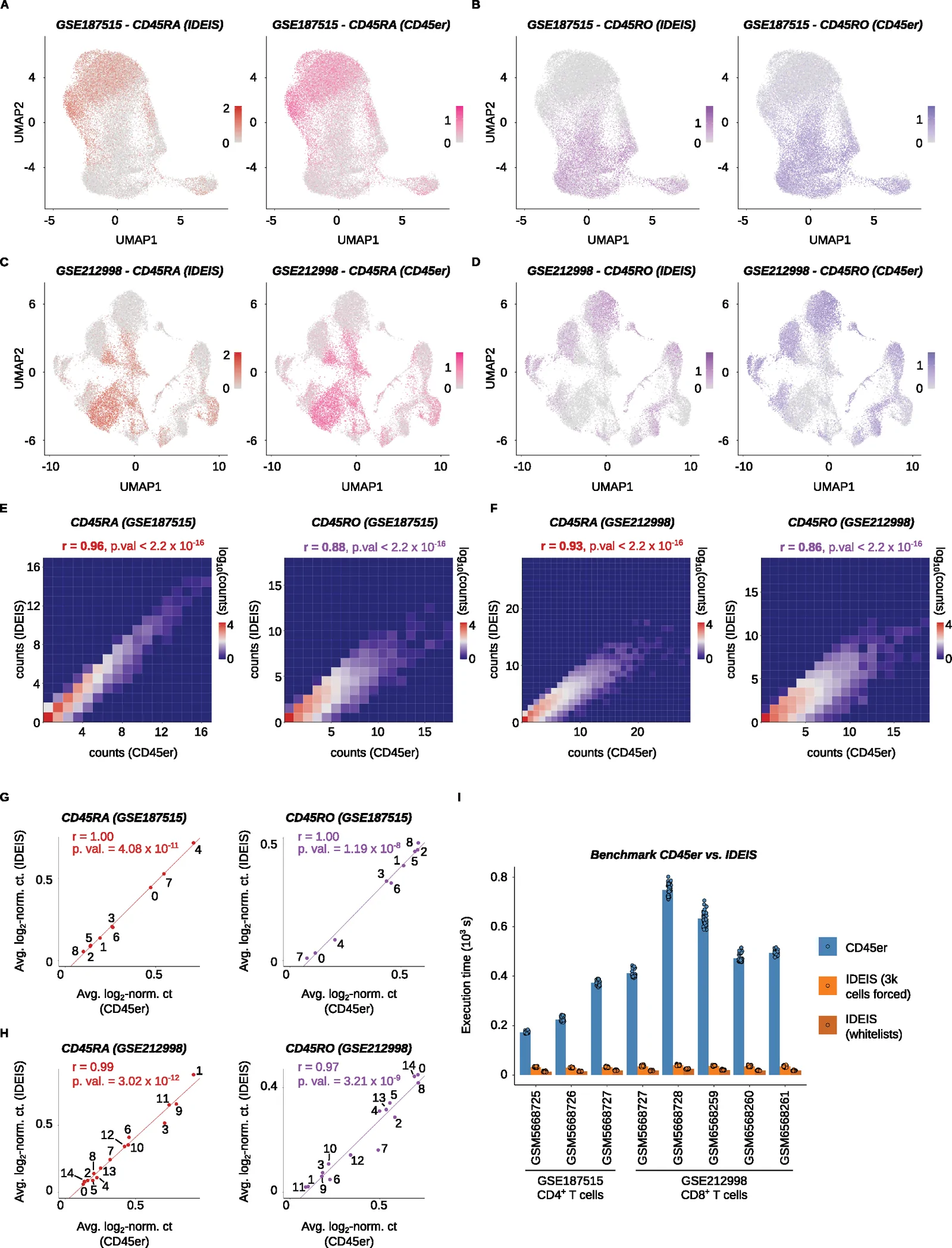
Benchmark between CD45er and IDES. **(A)** UMAP projections with log_2_-normalized counts of reads detected by IDEIS and log_2_-normalized sum of probabilities of reads matching *CD45RA* isoforms per cell. The comparison is shown for the Collora et al. dataset. **(B)** UMAP projections with log_2_-normalized counts of reads detected by IDEIS and log_2_-normalized sum of probabilities of reads matching *CD45RO* isoforms per cell. The comparison is shown for the Collora et al. dataset. **(C)** UMAP projections with log_2_-normalized counts of reads detected by CD45er and log_2_-normalized sum of probabilities of reads matching *CD45RA* isoforms per cell. The comparison is shown for the Yu et al. dataset. **(D)** UMAP projections with log_2_-normalized counts of reads detected by CD45er and log_2_-normalized sum of probabilities of reads matching *CD45RO* isoforms per cell. The comparison is shown for the Yu et al. dataset. **(E)** Tile plot of isoform counts detected by CD45er (x axis) and IDEIS (y axis) for *CD45RA* (left) and *CD45RO* (right). The counts per tile are shown in log_10_ scale with pseudo-count of 1. The plots are calculated for the Collora et al. dataset. r, Pearson’s correlation coefficient, p. val, p-value of correlation test between cell-wise isoform counts. **(F)** Tile plot of isoform counts detected by CD45er (x axis) and IDEIS (y axis, the values shown correspond to log_10_ with pseudo-count of 1) for *CD45RA* (left) and *CD45RO* (right). The counts per tile are shown in log_10_ scale with pseudo-count of 1. The plots are calculated for the Yu et al. dataset. r, Pearson’s correlation coefficient, p. val, p-value of correlation test between cell-wise isoform counts. **(G)** Linear regression and correlation of average log_2_-normalized sum of probabilities of read matching given isoform returned by CD54er per cell (x axis) and average log_2_-normalized counts of reads corresponding to given isoform by IDEIS (y axis), for the Collora et al. dataset. Each dot represents a value for cluster with numbering as shown on **Figure 2A**. The line represents linear regression curve. r, Pearson’s correlation coefficient, p. val, p-value for linear regression slope. **(H)** Linear regression and correlation of average log_2_-normalized sum of probabilities of read matching given isoform returned by CD54er per cell (x axis) and average log_2_-normalized counts of reads corresponding to given isoform by IDEIS (y axis), for the Yu et al. dataset. Each dot represents a value for cluster with numbering as shown on **Figure 3A**. The line represents linear regression curve. r, Pearson’s correlation coefficient, p. val, p-value for linear regression slope. **(I)** The comparison of execution times for CD45er and IDEIS on eight different samples from analyzed the Collora et al. and Yu et al. datasets. For each sample n = 40 runs were made. The dots represent the time execution of each separate run, the bar shows their average for given subset. In case of IDEIS the benchmark was done with forced number of cells on 3,000 (orange) or whitelist of cells (dark orange). Bottom numbers, dataset Accession Numbers, x axis labels, sample Accession Numbers.

In the next step, we compared the performance of IDEIS to that of a similar software called CD45er which was described previously (26). We analyzed the Collora et al. and Yu et al. datasets by IDEIS and CD45er and obtained comparable results (**Figures 6A–D**), as documented by a very strong correlation between isoform expression both at the single-cell level (**Figures 6E, F**) and at the cluster level (**Figures 6G, H**).

To compare the speed of CD45er and IDEIS, we benchmarked the datasets of Collora et al. and Yu et al. To obtain accurate results, we ran each software program 40 times and averaged the obtained execution times. IDEIS was tested in two different configurations: a) using whitelists generated from the initial datasets, which is faster, and b) forcing the estimated number of cells in data with any isoform to 3,000, which is used for further processing by salmon alevin; this method is slower because of exportation of matrix compatible with 10× format. To put both programs on an even starting ground, we used only reads from cells that passed the initial quality control checks. Because salmon alevin does not support multiple index generations in parallel, IDEIS was run on already prepared index files that were not included in the benchmarking time. However, the execution time of this step was negligible. In summary, IDEIS was 6×–29× faster than CD45er with the forcing-cells option and 11×–49× faster with the whitelist option than CD45er (**Figure 6I**).

### 3.7 3’ RNA sequencing method provides limited information on the CD45 splicing

**Figure 7:**
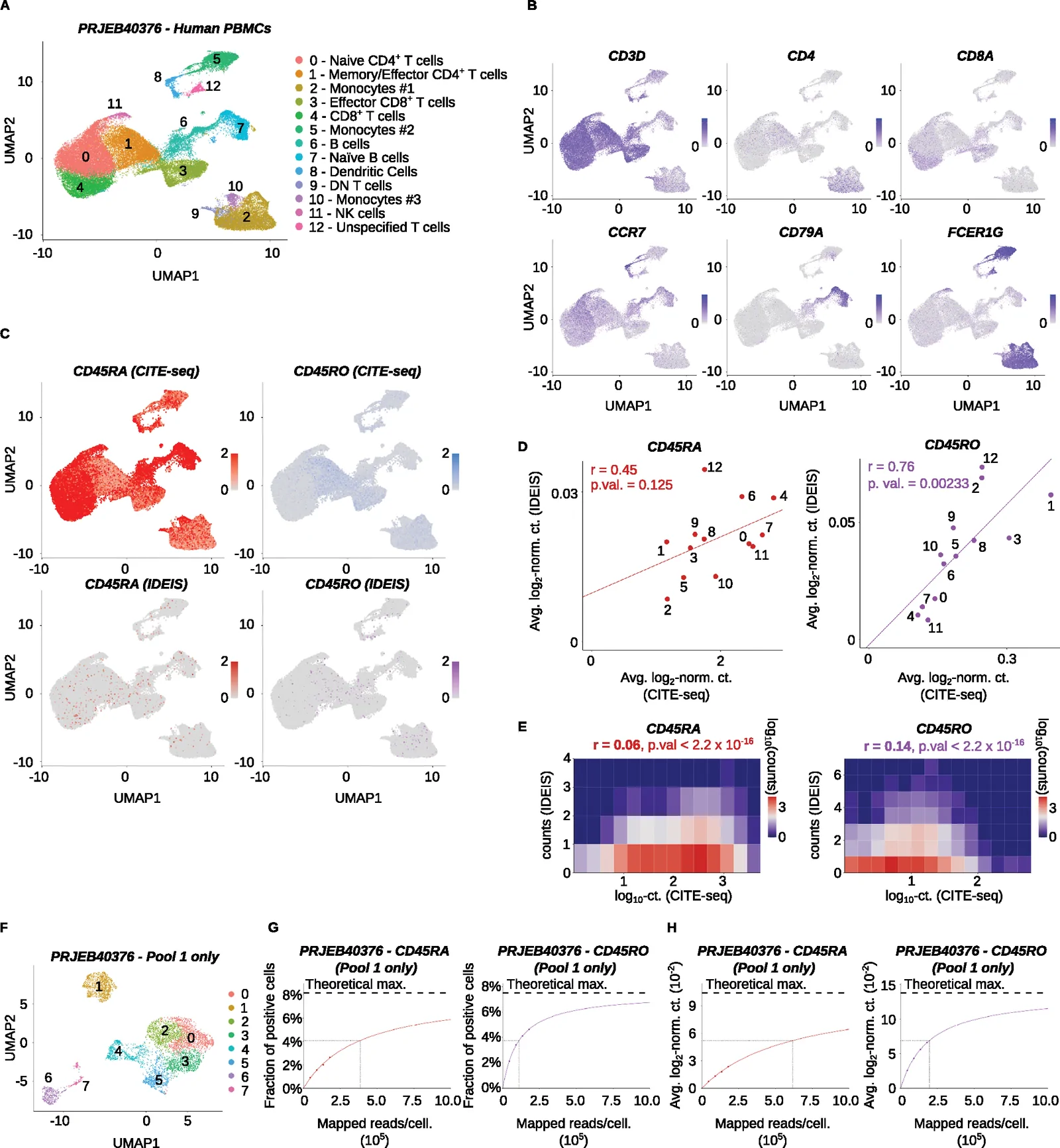
Analysis of CD45 isoforms of a human dataset generated from 3’ gene expression library. The Lawlor et al. dataset (PRJEB40376) was analyzed. **(A)** UMAP projection, clustering and expert annotations of the finalized dataset. The x and y axes show the first and second dimension of UMAP projection, respectively. **(B)** Visualization of log_2_-normalized expression of selected genes on UMAP projection of the dataset. **(C)** Log_2_-normalized counts of CITE-seq reads (top row) or reads detected by IDEIS software (bottom row) corresponding to *CD45RA* (left column) or *CD45RO* (right column) isoform on UMAP projection of analyzed dataset. To better visualize the expression, the maximum expression value was cut off at 2. **(D)** Linear regression and correlation of average log_2_-normalized CITE-seq read counts (x axis) or reads detected by IDEIS software (y axis) corresponding to *CD45RA* (dark red) and *CD45RO* (purple) isoforms for each cluster separately of the dataset. Each dot represents a value for cluster with numbering as shown on **(A)**. The line represents linear regression curve. r, Pearson’s correlation coefficient, p. val, p-value for linear regression slope. **(E)** Tile plot of isoform counts detected by CITE-seq (x axis) and IDEIS (y axis, the values shown correspond to log_10_ with pseudo-count of 1) for *CD45RA* (left) and *CD45RO* (right). The counts per tile are shown in log_10_ scale with pseudo-count of 1. r, Pearson’s correlation coefficient, p. val, p-value of correlation test between cell-wise isoform counts. **(F)** UMAP projection and clustering dataset from first pool of samples only. **(G)** The fraction of cells having a positive read count of indicated isoforms identified by IDEIS in function of estimated average number of mapped reads per cell, for *CD45RA* (dark red) and *CD45RO* (purple) isoforms, for Pool 1 of the Lawlor et al. dataset only. The dots show values calculated from subsampled dataset, full colored line shows the fit by two-parameter rational function, and black dashed line shows its asymptote representing maximum theoretically possible value. Black dotted lines show the point where 50% of maximum predicted value has been reached. **(H)** The evolution of average log_2_-normalized read count identified by IDEIS in function of estimated average number of mapped reads per cell, for *CD45RA* (red) and *CD45RO* (purple) isoforms, for Pool 1 of the Lawlor et al. dataset only. The meaning of graphical elements is the same as in **(G)**.

The previous analyses were run using input scRNA-seq data generated by Chromium Single Cell 5’ Reagent Kits. Such approaches are mainly used when TCR or BCR repertoire profiling is also analyzed, as most 3’ single-cell sequencing assays do not capture CDR3 sequences that lie closer to the 5’ end of receptor transcripts (30). To investigate whether 3’ sequencing data could serve as the input for IDEIS, we analyzed the scRNA-seq dataset of human PBMCs generated by Chromium Single Cell 3’ Library & Gel Bead Kit v2 for library (Accession Number PRJEB40376) (24) (**Figures 7A, B**). The comparison with CITE-seq showed that only limited information about the expression of *CD45RA* or *CD45RO* isoforms was obtained from the transcriptomic data by IDEIS (**Figure 7C**). On the level of individual clusters, *CD45RA* expression by IDEIS did not significantly correlate with the results of CITE-seq, while for the *CD45RO* isoform, there was a correlation between the two methods (**Figure 7D**). At the single-cell level, the results showed a very small, albeit significant, correlation in both cases, but the overall number of isoform reads per cell identified by IDEIS was very low (**Figure 7E**).

We investigated the effect of subsampling on the Lawlor et al. dataset. Due to size of the data, the analysis was limited to Pool 1 (**Figure 7F**, sample number SAMEA7463734; see **Supplementary Table 2**). The results showed that even with extensive sequencing, the maximum number of cells positive for either *CD45RA* or *CD45RO* remained below 10%. To reach the half-maximal performance of identification of *CD45RA* and *CD45RO* positive cells, the average sequencing depth would need to be 3.8 × 10^5^ and 1.1 × 10^5^ reads per cell, respectively (**Figures 7G, H**). Overall, this analysis showed that while we observed a limited correlation between CITE-seq and IDEIS in the case of the *CD45RO* isoform, the low overall number of isoform read counts per cell identified by IDEIS means that this tool has only limited use when employed to analyze 3’ sequencing data.

## 4 Discussion

IDEIS is an accurate tool for determining the splicing pattern of *CD45RA* vs *CD45RO* in CD4^+^ and CD8^+^ T cells using scRNA-seq data generated by the 5’ sequencing protocol. It can be used to determine and annotate particular T cell subsets without the need to use CITE-seq protocols with anti-CD45RA and anti-CD45RO antibodies, which reduces the length of the protocol and additional costs. Moreover, it could be used for the reanalysis of scRNA-seq experiments that have already been performed without the CITE-seq labeling. The recommended depth of sequencing for full IDEIS performance is estimated to be 15,000 reads per cell, which is typical in the field. The use of IDEIS for data generated by 3’ sequencing protocols is limited because of the rarity of reads mapping to the alternatively spliced exons in *PTPRC/CD45*, which are located towards the 5’ end of the transcript. However, if there is no alternative, IDEIS can still be used to retrieve information about *PTPRC/CD45* splicing, particularly concerning the CD45RO isoform.

Although not commonly used for detecting particular subsets, different types of immune cells have different *PTPRC/CD45* splicing patterns in mice (11). We used IDEIS to explore the *PTPRC/CD45* splicing patterns in murine leukocytes at the single-cell level. We identified that particular lymphocyte subsets can be identified by their *PTPRC/CD45* splicing patterns, such as B cells, by their expression of *CD45RA*, naïve and central memory T cells, NK cells by their expression of *CD45RB*, and effector memory T cells by their expression of *CD45RO*. The lack of CD45 CITE-seq data in murine immune datasets makes IDEIS a useful tool for subset annotation.

Haradhvala et al. previously developed CD45er, a script for the analysis of *PTPRC/CD45* splicing in scRNA-seq data to evaluate engineered T cells in B-cell lymphoma therapy (26). A comparison of this tool and IDEIS showed that, while both programs have similar results to human data, IDEIS has a substantial advantage for third-party users. First, IDEIS uses a faster algorithm than CD45er does. Second, IDEIS is much easier to use with murine data than CD45er. Third, the results generated by IDEIS are easier to use, as they are exported as MTX objects that can be loaded in R for downstream analysis. If the user opts to use the Seurat object as an input, the results of the analysis are added directly to it as a new assay, making further analyses even easier.

One of the main drawbacks of this method is the limited applicability of data generated from 3’ gene expression libraries. The analysis of such data shows that there is, at the cluster level, a limited correlation between the results obtained by CITE-seq and IDEIS in the case of the *CD45RO* isoform. However, the correlation at the single-cell level between the results of the two methods was very small, albeit statistically significant. Moreover, the overall isoform read count obtained by IDEIS is very low, meaning that even a small noise might potentially perturb the analysis, thereby limiting the usefulness of IDEIS for the study of such data. However, this is an inherent property of 3’ RNA library preparation (30), leading to the fact that information about *PTPRC/CD45* splicing is barely present in the final data. However, 5’-RNA sequencing is often used for T-cell scRNA-seq experiments due to its compatibility with parallel VDJ repertoire sequencing. Another caveat of IDEIS is its inability to use biological information that can be present in the first read with a cellular barcode and unique molecular identifier, as well as its dependency on read lengths; however, these points have no significant impact on the global performance of the software; in particular, the read length dependency is mitigated by salmon alevin’s split of weight for multi-mapping reads.

We did not analyze the role of the length of the sequencing reads because of the scarcity of required data. We assume that longer reads should improve the quality of the results returned by IDEIS; however, this assumption requires further confirmation. We show here that even relatively short reads (26 and 98 bp for R1 and R2, respectively, used in the Collora et al. dataset) are sufficient for analysis. Therefore, IDEIS should perform well in most sequencing setups.

Overall, IDEIS is a tool for the fast analysis of *PTPRC/CD45* isoform splicing, facilitating the interpretation of scRNA-seq data of human and mouse lymphocytes. Its ease of use makes it suitable for use even by scientists with limited experience in single-cell data analysis.

## Data Availability

Publicly available datasets were analyzed in this study. The software is available at https://github.com/Lab-of-Adaptive-Immunity/IDEIS. The code used to process the downloaded datasets, prepare figures as well as running benchmarking is available at: https://github.com/Lab-of-Adaptive-Immunity/IDEIS_data_analysis. The data used for analyses are available on public databases under their respective accession numbers (see **Supplementary Tables 1**, **2**). These data can be downloaded using scripts provided in the link above.
