# Correction: IDEIS: a tool to identify PTPRC/CD45 isoforms from single-cell transcriptomic data

**DOI:** 10.3389/fimmu.2026.1952440

**Published:** 2026-08-12

**Authors:** Juraj Michalik, Veronika Niederlova, Ondrej Stepanek

**Affiliations:** Laboratory of Adaptive Immunity, Institute of Molecular Genetics of the Czech Academy of Sciences, Prague, Czechia

**Keywords:** PTPRC, CD45, isoform, alternative splicing, immunology, T cell, single-cell RNA sequencing, gene expression

An incorrect number was provided for “Czech Science Foundation”. The correct number is “22-18046S”. The **Funding** statement has been corrected to read:

“The author(s) declare that financial support was received for the research, authorship, and/or publication of this article. This project was supported by the National Institute of Virology and Bacteriology (Programme EXCELES, LX22NPO5103 to OS), funded by the European Union—Next Generation EU, and the Czech Science Foundation (22-18046S to OS).”

The original version of this article has been updated.

