# Supplementary material for: IDEIS: a tool to identify PTPRC/CD45 isoforms from single-cell transcriptomic data
Source: Front Immunol. 2024 Oct 9;15:1446931. doi: 10.3389/fimmu.2024.1446931 (PMC11496083; doi:10.3389/fimmu.2024.1446931)
Supplement: Supplementary file 1 [file DataSheet1.pdf]

# *Supplemental Material*

## 1 Supplemental Tables

**Supplemental Table 1:** List and details of analyzed data sets.

**Supplemental Table 2:** Details on samples analyzed for each data set.

# 2 Supplemental Figures

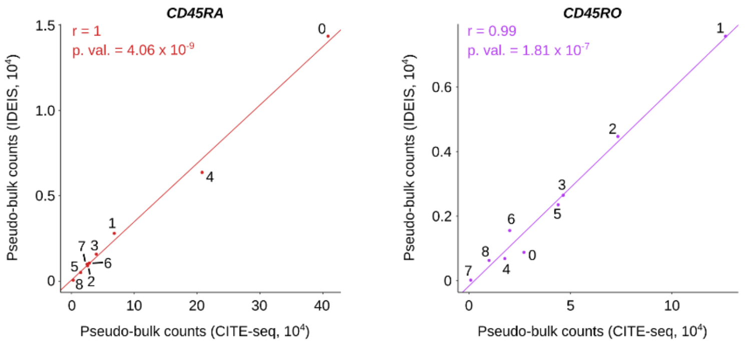


**Supplemental Figure 1: Linear regression on pseudo-bulk data of *PTPRC/CD45* isoforms of CD4$^{+}$ T cells from Collora *et al.* data set (GSE187515).**

Linear regression and correlation of pseudo-bulk CITE-seq read counts (x axis) or counts of reads detected by IDEIS software (y axis) corresponding to *CD45RA* (dark red) and *CD45RO* (purple) isoforms for each cluster of the data set. Each dot represents a value for cluster with numbering as shown on (A). The line shows linear regression curve. r – Pearson's correlation coefficient, p. val – p-value of slope.

## 3 Technical Note

This note specifies the details about IDEIS pipeline upon the execution of the software as described on Figure 1B. For details about how to run IDEIS, all its options and its output please refer to the GitHub Page (https://github.com/Lab-of-Adaptive-Immunity/IDEIS).

**List of exons for CD45/PTPRC:** list of CD45/PTPRC exons in multi-FASTA format. Example excerpt:

```
>Ptprc-R R
TTTGTTCTTAGGGTAAGAGAGTAGGAAACTTGCTCCCCATCTGATAAGACAGAGTGCAAA
GGAGACCCTATTTCTTAGGGGCACAGCTGATCTCCAGATATGACCATGGGTTTGTGGCTC
AAACTTCTGGCCTTTGGATTTGCCCTTCTGGACACAGAAGTCTTTGTCACAG
GGCAAACACCTACACCCAGTGATG

>Ptprc-A A
AACTGAGCACAACAGAGAATGCCCTTCTTCTGCCTCAAAGTGACCCCTTACCTGCTCGCA
CCACTGAATCCACACCCCCAAGCATCTCTGAAAGAGGAAATGGCTCTTCAGAGACCACAT
ATCATCCAG
```

**Transcript building rules:** rules indicating what exons each reference sequence contains and which is an exon of interest for given reference. Example:

```
A:R,A,B,C,O:Ptprc-RABCO-1:Ptprc-RA
```

There are 4 fields separated by ':'. The first field indicates **the element of interest**, which is exon A in this case (this field might be a junction, in such case this field is empty). The second field indicates how exons or sequences are chained – here R is followed by A, B, then C and finally by sequence O. If the element of interest is a junction, it is indicated by '-'. The last two fields indicate respectively the name of transcript and gene as used by salmon, so last field should have identical name for sequences tracing down same isoforms (such as *CD45RA*).

**10X-produced BAM file:** BAM generated by analysis by 10X Cell Ranger. Generated by 'cellranger count' tool. Please refer to Cell Ranger manual for more details.

**Filtering to CD45 + conversion to FASTQ using 10X bamtofastq:** selection of reads mapping to *PTPRC* locus and their conversion to FASTQ file. This is done by this command:

```
./bamtofastq-1.4.1 bam_path --locus=gene_range fastq_path
```

where:

- *bam_path* specifies the path to **10X-produced BAM file**;
- *locus* specifies the range to which reads will be filtered (it is sufficient that read partially overlaps the interval); the interval depends on species and reference;
- *fastq_path* is the path where the results of 10X bamtofastq will be saved.

The main result of this command used further are **Paired (R1, R2) FASTQ files**.

**Reference construction** builds the reference from **List of exons for CD45/PTPRC** using **Transcript building rules.** This is done the following way:

- The sequence of element of interest is taken (none in case of junction);
- The flanking sequences around this sequence are built based on Transcript building rules (which indicate the preceding/following exons and their order), which are trimmed based on length of input reads so the flanks are actually shorter than reads – this means completely mapping read has to at least partially overlap the element of interest.

Once these sequences are created, the following steps take place:

- The indexing of generated reference sequences, which is done by command:

  ```
  salmon index -t transcriptome_path –i reference threads
  ```

  where:

  - *transcriptome_path* is the path to the previously created reference sequences;
  - *reference* is the path where final reference is stored.

- Generation of *tgMap.tsv* file, which is stored in directory pointed by *reference* and links gene and transcriptome names.

At the end of this step the **Reference of CD45/PTPRC isoforms** is created. The step is skipped over if the reference already exists.

**Mapping to reference with Salmon Alevin** maps the reads present in **Paired (R1, R2) FASTQ files** to **Reference of CD45/PTPRC isoforms.** This is done by the command:

```
salmon alevin -l library -1 fastqs_R1 -2 fastqs_R2 –i reference -o target_path protocol --tgMap tgmap cells_to_look –dumpMtx -p threads
```

where:

- *library* is a type of sequencing (ISF in case of 5' sequencing, ISR for 3' sequencing);
- *fastqs_R1* and *fastqs_R2* are reads R1 and R2 from **Paired (R1, R2) FASTQ files**;
- *reference* is the path to **Reference of CD45/PTPRC**;
- *target_path* is the path to output;
- *protocol* is the type of protocol, by default Chromium;
- *tgmap* is the path to tgmap created during indexing;
- *cells_to_look* either contains number of expected cells, number of forced cells, or a whitelist of cells either passed as option to IDEIS or generated from data set passed as an input to IDEIS as well;

- *threads* specifies number of threads, defaults to 1.

All of these variables are handled implicitly within the IDEIS with options controlling parameters. The main output of this step are the **Counts of CD45/PTPRC isoforms.**

**Conversion to Seurat object with R/Seurat** is done using R and Seurat package. If --*data-set* option was used, the small *Extract_whitelist_from_rds.R* file is run to extract barcodes from data set with following command:

```
Rscript --no-restore Rscript_path data_set whitelist_path
```

where:

- *Rscript-path* specifies the path to *Extract_whitelist_from_rds.R* file;
- *data_set* is the path to data set serving as an input;
- *whitelist_path* is the final destination of extracted barcode list.

After this step, the file runs another script which runs a file *Generate_final_mtx.R* that creates final output files:

```
Rscript --no-restore Rscript_path input_path count_output_path
main_parameters.sequencing_type whitelist data_set data_set_path
```

where:

- *Rscript-path* specifies the path to *Generate_final_mtx.R* file;
- *input_path* is a path to directory with results (that is passed to IDEIS) where the remainder of IDEIS output is stored;
- *count_output_path* is a path to result sub-directory where counts are stored;
- *main_parameters.sequencing_type* is a type of sequencing (passed to IDEIS);
- *whitelist* is path to whitelist; used only if --*whitelist* option was passed IDEIS;
- *data_set* is path to data set; used only if --*data_set* option was passed IDEIS;
- *data_set_path* is path to result sub-directory where final data set will be stored; used only if --*data_set* option was passed to IDEIS;

The variables are handled implicitly by pipeline unless explicitly stated that they are defined by IDEIS options.

Once the pipeline is finished, the **Results** directory is generated, with the main output being **RDS of isoform count matrix (sparse matrix).** If --*data_set* was passed as parameter, this count matrix is directly added to provided data set as assay.
